# Real-Time Reconstruction of the Eddy-Current Magnetic Field in a Local Region of a Maneuvering UUV Platform

**DOI:** 10.3390/s26216945

**Published:** 2026-11-03

**Authors:** Peng Gu, Dong Gao, Chunjiang Bian, Minghui Zhu

**Affiliations:** 1 Key Laboratory of Electronics and Information Technology for Space Systems, National Space Science Center, Chinese Academy of Sciences, Beijing 100190, China; gupeng19@mails.ucas.ac.cn (P.G.); bianchunjiang@nssc.ac.cn (C.B.); zhuminghui20@mails.ucas.ac.cn (M.Z.); 2 University of Chinese Academy of Sciences, Beijing 100190, China

**Keywords:** eddy-current magnetic field, magnetic interference compensation, low-dimensional spatial basis, step response, differential measurement, unmanned underwater vehicle

## Abstract

When an unmanned underwater vehicle (UUV) with an aluminum-alloy hull maneuvers, its motion through the geomagnetic field generates an eddy-current magnetic field that interferes with the onboard magnetometer. We propose a method that extracts a low-dimensional spatial basis offline and reconstructs the eddy-current field within a local region online with two magnetometers. Offline, transient finite element method (FEM) simulations with unit excitations along the three body axes provide a quasi-static mode and a relaxation mode per excitation channel together with their step responses. Online, the time derivative of the primary magnetometer output drives a feed-forward reconstruction through the step responses, and the differential measurement of a single auxiliary magnetometer gives one amplitude factor, estimated sample by sample, that corrects the amplitude deviation of the offline model. The method requires neither angular-rate measurements nor calibration maneuvers. Six spatial modes represent the field of yaw, pitch, and roll maneuvers over the entire sensor candidate region with an error below 0.8%, and that of a combined reversing maneuver exciting all three channels with an error of 1.2%. With five classes of magnetometer error and two classes of model error injected, the region reconstruction error is 2.7–3.2% under a favorable installation and 6.1–7.3% under a typical installation. The residual at the primary magnetometer, the eddy-current interference remaining after vector compensation, is 8–9 nT and 14–15 nT. Open-loop feed-forward reconstruction yields 9.7–10.9%, and the complete Tolles–Lawson model calibrated on six other maneuvers yields 9–10 nT and 13–14 nT. In a further test, an independent FEM model with a 10% lower conductivity, an added conductor, and a new mesh generates the data, while the method keeps the original offline model. The amplitude factor converges to 0.90, the true ratio between the fields of the two models, and the residual stays within 4–10 nT. After the primary magnetometer is relocated, the residual without recalibration is 10 nT for the proposed method and 25–40 nT for the Tolles–Lawson model calibrated at the original position.

## 1. Introduction

Geomagnetic navigation and magnetic survey tasks require high-precision magnetometers to be carried on underwater platforms, and the magnetic interference produced by the platform itself is the primary limitation on measurement accuracy [1]. The magnetic interference of a maneuvering platform is conventionally divided into three components, namely the permanent, induced, and eddy-current magnetic fields [2]. Vector calibration procedures for the first two components are well established [3,4,5,6,7,8]. The eddy-current magnetic field is induced in the conductive structure as the platform moves through the geomagnetic field. It is proportional to the rate of change of the geomagnetic vector in the body frame, appears only during maneuvers, and is usually regarded as small [4,9]. At the ship scale, however, the eddy-current field is the second-largest signature source after the ferromagnetic effect, and non-magnetic conductive materials such as aluminum and stainless steel also produce a distinct eddy-current signature [10,11,12]. For an unmanned underwater vehicle (UUV) with an aluminum-alloy hull, the hull itself is non-magnetic, and the permanent and induced fields originate mainly from the equipment inside the hull, so they can be removed by vector calibration [3,4,13]. The hull nevertheless produces a significant eddy-current field during maneuvers [11], which becomes the dominant residual interference after calibration. This residual cannot be removed by filtering. Low-pass filtering has been applied on the assumption that the eddy-current field consists of high-frequency components [13]. The eddy-current field, however, follows the rate of change of the geomagnetic vector and therefore varies at the maneuver frequency of 0.1–1 Hz, the band in which the measured geomagnetic signal also varies during the maneuver. Consequently, a filter that removes the interference would also remove this signal.

Existing treatments of the eddy-current magnetic field follow three approaches. In the first approach, some studies of underwater magnetic vector compensation neglect the eddy-current field in the model on the grounds that the rate of change of the geomagnetic vector is low at low speed [3,4,9]. Others treat it as measurement noise and remove it by low-pass filtering [6,13]. Neither premise holds during attitude maneuvers. In the second approach, aeromagnetic compensation describes the eddy-current field with the Tolles–Lawson (T-L) model, in which the time derivative of the magnetometer output serves as the regressor. This model has evolved from eight eddy-current coefficients [2] to extended models with additional interference terms [14] and to a 3 × 3 coefficient matrix with online updating [15,16,17]. Its derivation and calibration procedure are summarized in Ref. [18], and data-driven compensation models trained on flight data have been developed for magnetic navigation [19]. Real-time implementations on small unmanned aircraft [20] and the effect of attitude variations on data quality [21] have also been reported. However, the online updating of the coefficients relies on a reference for the geomagnetic vector, which is provided by attitude information [15] or by the scalar total-field constraint [17]. A fixed-parameter filter driven by the history of the geomagnetic vector in the platform frame has been applied in the same spirit to the motion noise of sensors carried by autonomous underwater vehicles [22]. The coefficients of the T-L model are identified from calibration maneuvers and describe only the measurement distortion at the installation point of the magnetometer. Consequently, whenever candidate installation positions are compared at the design stage, or the position is changed together with the payload between missions, each position must be recalibrated. In the third approach, the ship-signature community performs high-fidelity offline forward computations and facility measurements of eddy currents [10,12,23], and statistical prediction of signatures from hull parameters has been explored [24]. These provide the spatial distribution of the eddy-current field, but a single finite element method (FEM) solution takes hours [23,25], and the differences between the model and the real structure in conductivity, weld seams, and geometric details enter the computed results directly as errors [12].

The problem left unsolved by all three approaches is that the spatial distribution and the amplitude of the eddy-current magnetic field cannot be obtained online at the same time. The eddy-current field is produced by sources distributed over the entire conductive structure, and the field at any position is determined jointly by the transient response of the structure and by the position itself. The T-L model holds only at the calibration point and contains no spatial structure. The offline forward computation contains the spatial structure but cannot be updated in real time, and its amplitude is governed by the discrepancy between the model and the real structure. Therefore, reconstructing the space–time distribution of the eddy-current field within a local region online, while keeping its amplitude independent of the absolute accuracy of the offline model, remains an open problem.

To address this problem, we propose a real-time reconstruction method for the eddy-current magnetic field. A low-dimensional spatial basis and the step responses of its modes are extracted offline from transient FEM simulations with unit excitations along the three body axes. Online, the rate of change of the primary magnetometer output serves as the excitation for a feed-forward reconstruction, and the differential measurement of a single auxiliary magnetometer provides an online estimate of an amplitude factor that corrects the amplitude deviation between the offline model and the real platform.

Both elements of this method have precedents. Snapshot-based model order reduction provides low-dimensional spatial bases for transient eddy-current problems [26,27,28], but these bases have been used to accelerate forward computation rather than for online reconstruction from magnetometer data. In fluid dynamics, the gappy proper orthogonal decomposition reconstructs an unsteady field from a few sensors by solving for the coefficients of a precomputed basis [29], and sensor locations for such reconstructions are selected from the basis itself [30]. Likewise, the deployment region of the magnetometers of a closed-loop degaussing system has been chosen from computed variations of the signature [31]. Differential measurements with a pair of magnetometers have been used to cancel platform interference in geomagnetic vector measurement [3] and on small satellites [32]. In the latter application, the ratio of the interference at the two sensors is estimated from the data under the assumption that the interference is uncorrelated with the ambient field. Attitude information has also been used to suppress the interference components [33]. These schemes estimate the interference itself rather than the amplitude of an offline spatial model. Combining the precomputed spatial basis of a reduced model with an online amplitude correction from a single differential measurement has not been attempted for the eddy-current field.

The main contributions of this work are as follows:

(1) We use transient FEM data from three unit excitations along the body axes and from four independent maneuvers, including one in which the three excitation channels act simultaneously. With these data, we verify that the eddy-current magnetic field of the platform within the sensor candidate region is spanned by six maneuver-independent spatial modes. We also verify that the response of each mode coefficient to an arbitrary excitation is fully determined by its step response.
(2) We formulate a differential measurement model referenced to the primary magnetometer. With the steady-attitude interval before the maneuver as the offset reference, one amplitude factor is estimated sample by sample from the differential measurement of a single auxiliary magnetometer. The method requires neither angular-rate measurements nor calibration maneuvers, and both the systematic errors of the magnetometers and the amplitude error of the offline model enter the result only through the amplitude factor.
(3) We construct an error budget from parameters given in material handbooks and device datasheets. In cases in which magnetometer errors and model errors are injected simultaneously, we compare the proposed method with the T-L model, open-loop feed-forward reconstruction, and full-coefficient estimation in terms of two metrics, namely the region reconstruction error and the residual at the primary point.

The remainder of this paper is organized as follows. Section 2 establishes the low-dimensional mode structure of the eddy-current field and the time response of the mode coefficients. Section 3 presents the acquisition of the excitation, the feed-forward reconstruction, the differential measurement, and the estimation of the amplitude factor. Section 4 verifies the mode library, compares the methods, and evaluates the error budget using transient FEM simulations. Section 5 concludes the paper.

## 2. Eddy-Current Magnetic Field of a Maneuvering Platform

### 2.1. Generation of the Eddy-Current Field and Its Characteristic Time

This study considers a UUV with an aluminum-alloy hull. Its load-bearing structures, including the hull, the stiffening rings, and the lifting lugs, are made of non-magnetic aluminum alloy with a relative permeability close to unity. The body frame O-xyz is defined such that the *x* axis lies along the longitudinal axis and points aft, the *y* axis points vertically upward when the vehicle is level, and the *z* axis follows the right-hand rule.

The eddy-current field is generated as follows. When the platform maneuvers, the geomagnetic vector expressed in the body frame changes with the attitude. The magnetic flux through every closed loop of the conductive structure changes accordingly and induces eddy currents inside the structure. The additional field produced by these currents is superimposed on the readings of the magnetometers inside the hull. This additional field is the eddy-current magnetic field, and its amplitude is proportional to the speed of motion and to the electrical conductivity [34].

The temporal behavior of the eddy-current field is governed by the characteristic time of the structure. For a thin-walled conductive loop such as the hull, whose eddy-current behavior in transverse and axial fields has been analyzed for cylindrical shells [35], the characteristic time for the field to penetrate the wall can be estimated from an equivalent resistance–inductance (RL) model to be on the order of $\tau_{\mathrm{est}}\approx \frac{\mu_{0}\sigma R_{h}t_{h}}{2}$ [23], where $\mu_{0}$ is the vacuum permeability, σ is the electrical conductivity, $R_{h}$ is the hull radius, and $t_{h}$ is the wall thickness. The effective decay time constant is determined from the transient simulation data. Let Ω denote the angular rate of the maneuver. When Ωτ≪1, the eddy-current distribution follows the excitation quasi-statically, and the relaxation appears as a first-order correction of order Ωτ.

### 2.2. Low-Dimensional Mode Structure and Time Response of the Coefficients

In the body frame, the geometry of the platform is fixed, and the only time-varying input during a maneuver is the projection $B_{0}\left(t\right)$ of the geomagnetic vector onto the body frame. Over the scale of the platform and the duration of a single maneuver, the geomagnetic field can be regarded as uniform and constant, so the rate of change of $B_{0}\left(t\right)$ is determined by the angular velocity of the platform:

(1) $$u\left(t\right):={\dot{B}}_{0}\left(t\right)=-\omega \left(t\right)\times B_{0}\left(t\right)$$

where ω(t) is the angular velocity expressed in the body frame, and u(t) is referred to as the excitation vector, whose three components correspond to three excitation channels.

The relation between the eddy-current field and the excitation is linear and dynamic. The permeability of aluminum is close to that of vacuum, and its conductivity does not depend on the field, so the field equations are linear in the sources and the eddy-current field obeys superposition with respect to the excitation [35]. For a fixed geometry and material, the eddy-current equations discretized by the finite element method form a linear dynamic system in state-space form [26], and the low-order reduced representation of such a system is an established practice in transient electromagnetic computation [26,27,28]:

(2) $$M_{e}\dot{x}\left(t\right)+Kx\left(t\right)=\sum_{k=x,y,z}f_{k}\;u_{k}\left(t\right)$$

where x is the discretized electromagnetic state, $u_{k}\left(t\right)$ is the *k*-th component of the excitation vector of Equation (1), and $M_{e}$, K, and $f_{k}$ depend only on the geometry, material, mesh, and boundary conditions. The eddy-current field in the cavity inside the hull is a linear output of the state, b(r,t)=C(r)x(t). The system matrices remain unchanged during a maneuver. We expand the response in the generalized eigenmodes of Equation (2). Each eigenmode decays as a first-order system with its own time constant, and the single-loop RL model of Ref. [23] is the one-mode case. Denoting the spatial mode (hereafter referred to as the mode) of the *n*-th eigenmode under the *k*-th excitation channel by $g_{kn}\left(r\right)$ and its amplitude by $q_{kn}\left(t\right)$, we obtain

(3) $$b\left(r,t\right)=\sum_{k=x,y,z}\;\sum_{n\geq 1}g_{kn}\left(r\right)\;q_{kn}\left(t\right),\;\tau_{n}{\dot{q}}_{kn}\left(t\right)+q_{kn}\left(t\right)=u_{k}\left(t\right)$$

where $\tau_{n}$ is the time constant of the *n*-th eigenmode, and the excitation strength of each eigenmode has been absorbed into the definition of the mode.

For low-frequency maneuvers, the expansion reduces to a small number of maneuver-independent modes. When all eigenmodes with a noticeable contribution satisfy $\Omega \tau_{n}\ll 1$, the amplitude can be expanded as $q_{kn}\approx u_{k}-\tau_{n}{\dot{u}}_{k}$. Substituting this expansion into Equation (3) and collecting the terms in $u_{k}$ and ${\dot{u}}_{k}$ yields

(4) $$b\left(r,t\right)\approx \sum_{k=x,y,z}\left[G_{k}\left(r\right)\;u_{k}\left(t\right)-h_{k}\left(r\right)\;{\dot{u}}_{k}\left(t\right)\right]$$

where $G_{k}=\sum_{n}g_{kn}$ is the quasi-static mode of channel *k*, and $h_{k}=\sum_{n}\tau_{n}g_{kn}$ is the relaxation mode of the same channel. Both modes depend only on the geometry and the material and are independent of the maneuver history. Numbering the retained modes as $g_{1},\cdots ,g_{N_{m}}$ with the corresponding amplitudes $\alpha_{1}\left(t\right),\cdots ,\alpha_{N_{m}}\left(t\right)$, we approximate the eddy-current field by

(5) $$b\left(r,t\right)\approx \sum_{m=1}^{N_{m}}g_{m}\left(r\right)\;\alpha_{m}\left(t\right)$$

Each of the three excitation channels produces one quasi-static mode and one relaxation mode, so that $N_{m}\leq 6$ to first order; the actual number is determined from the data.

The amplitude equation in Equation (3) is linear and time-invariant, so the response of each mode coefficient to an arbitrary excitation is given by Duhamel’s integral, the convolution of the excitation with the derivative of the step response. Denoting the response of the coefficient of the *m*-th retained mode to a unit step excitation in channel *k* by $S_{km}\left(t\right)$, we have

(6) $$\alpha_{m}\left(t\right)=\sum_{k=x,y,z}\int_{0}^{t}{\dot{S}}_{km}\left(t-\xi \right)\;u_{k}\left(\xi \right)\;d\xi$$

The step response $S_{km}\left(t\right)$ is extracted directly from three transient solutions with a unit excitation along each body axis and fitted with a multi-exponential function on a fixed grid of time constants, $S_{km}\left(t\right)=\sum_{i}w_{kmi}\left(1-e^{-t/\tau_{i}}\right)$. Equation (6) then reduces to the recursion of a set of first-order linear filters, which integrates a piecewise-linear input exactly. Equation (4) is the first-order truncation of Equation (6) for Ωτ≪1 and is used to determine the number of modes, whereas the online computation uses Equation (6).

## 3. Online Reconstruction Method

### 3.1. Acquisition of the Excitation and Feed-Forward Reconstruction

A total of *M* three-axis magnetometers are installed inside the hull at positions $r_{j}$ (j=0,1,⋯,M−1). The magnetometer at $r_{0}$ is the primary magnetometer, that is, the sensor used for geomagnetic navigation, and its location is hereafter referred to as the primary point. The output of the *j*-th magnetometer is

(7) $$y_{j}\left(t\right)=B_{0}\left(t\right)+b\left(r_{j},t\right)+n_{j}\left(t\right)$$

where $n_{j}\left(t\right)$ is the measurement noise.

The excitation can be obtained from the output of the primary magnetometer itself. Equation (6) shows that, once the excitation u(t) is known, the mode coefficients follow directly from the step responses without any inversion. The output of the primary magnetometer is dominated by $B_{0}\left(t\right)$, and the eddy-current field accounts for only a fraction on the order of $10^{-3}$. Therefore, $u={\dot{B}}_{0}$ can be obtained from the finite difference in time of the primary magnetometer output, and no angular-rate measurement is required:

(8) $$\hat{u}\left(t\right)=\frac{d}{dt}\left[y_{0}\left(t\right)-\hat{b}\left(r_{0},t\right)\right]$$

where $\hat{b}\left(r_{0},t\right)$ is the current estimate of the eddy-current field at the primary point, which removes the rate of change in the eddy-current component itself. The first pass takes $\hat{b}=0$; the reconstruction is then substituted back into Equation (8), and three passes are used. The derivative is evaluated as the central difference over one sampling interval. After differencing, the measurement noise is on the order of $\sigma_{n}\sqrt{2}/\Delta t$, which represents a perturbation on the order of $10^{-3}$ relative to the magnitude of u during a maneuver, about $10^{4}$ nT/s.

Substituting $\hat{u}$ into Equation (6) yields the coefficients ${\hat{\alpha }}_{m}\left(t\right)$, and the eddy-current field at any position within the candidate region is reconstructed by Equation (5). This result is hereafter referred to as the feed-forward reconstruction and is denoted by ${\hat{b}}_{f}\left(r,t\right)$.

The accuracy of the feed-forward reconstruction is limited by the absolute amplitude of the offline model. The conductivity, the weld seams and contact resistance, and the geometric simplification all cause the amplitude of the real eddy-current field to deviate from that of the model. The part of this deviation that acts on all modes in the same proportion is referred to as the amplitude scaling error and is denoted by $\epsilon_{s}$. The primary magnetometer alone cannot identify this deviation, because the eddy-current component in its output is inseparable from $B_{0}$.

### 3.2. Differential Measurement and Online Estimation of the Amplitude Factor

The difference between the outputs of the auxiliary and primary magnetometers provides a direct measurement of the amplitude of the eddy-current field. With the primary magnetometer as the reference, the difference for each remaining magnetometer is formed as

(9) $$d_{j}\left(t\right):=y_{j}\left(t\right)-y_{0}\left(t\right)=b\left(r_{j},t\right)-b\left(r_{0},t\right)+n_{j}\left(t\right)-n_{0}\left(t\right)$$

Under ideal alignment and synchronization, $B_{0}\left(t\right)$ cancels completely in the difference, which then contains only the spatial difference in the eddy-current field.

The offset of the difference is determined before the maneuver. Before the maneuver begins, the attitude of the platform is stable, and the eddy-current field decays to zero within a few multiples of τ. The time average of the difference during this period is a constant unrelated to the maneuver. It consists of the difference between the permanent fields at the two positions and the constant part, in this attitude, of systematic errors such as axis misalignment and scale-factor mismatch. This constant is taken as the pre-maneuver offset and subtracted from all subsequent differences.

The amplitude factor is estimated sample by sample from the differential measurement. Let the real eddy-current field be the product of the feed-forward reconstruction and an unknown factor κ. Stacking the M−1 differences into a column vector gives

(10) $$d\left(t\right)=\kappa \;\psi \left(t\right)+\varepsilon \left(t\right),\;\psi \left(t\right)={\left[{\hat{b}}_{f}\left(r_{j},t\right)-{\hat{b}}_{f}\left(r_{0},t\right)\right]}_{j=1,\cdots ,M-1}$$

where ψ(t) is the difference of the feed-forward reconstruction between each auxiliary location and the primary point, and ε(t) is the differential noise. All difference channels share the noise $n_{0}$ of the primary magnetometer. If the magnetometer noises are mutually independent with a single-axis variance of $\sigma_{n}^{2}$, the noise covariance of the stacked difference is

(11) $$R=\sigma_{n}^{2}\left[\left(I_{M-1}+11^{T}\right)⊗I_{3}\right]$$

Equation (10) contains a single unknown. At each sample, the 3(M−1) scalar equations are solved with the weighting given by R:

(12) $$\hat{\kappa }\left(t\right)=\frac{\psi^{T}\left(t\right)\;R^{-1}d\left(t\right)}{\psi^{T}\left(t\right)\;R^{-1}\psi \left(t\right)}$$

A single auxiliary magnetometer (M=2) is sufficient to identify κ. The sample-by-sample solution makes no assumption about the time variation in κ, whereas accumulating the numerator and the denominator over time yields the recursive solution for a constant κ.

Equation (12) contains one unknown, so its condition number is one and no ill-conditioning arises. With the covariance of Equation (11), the variance of the estimate is $\mathrm{Var}\left(\hat{\kappa }\right)=\frac{1}{\psi^{T}R^{-1}\psi }$, which equals $\frac{2\sigma_{n}^{2}}{{∥\psi ∥}^{2}}$ for M=2. The estimation error δκ enters Equation (13) as $\delta \kappa \;{\hat{b}}_{f}\left(r_{0},t\right)$, whose root-mean-square (RMS) value per component is $\sqrt{\frac{2}{3}}\;\sigma_{n}\frac{∥{\hat{b}}_{f}\left(r_{0},t\right)∥}{∥\psi (t)∥}$. Because ψ and ${\hat{b}}_{f}$ are produced by the same excitation through the same step responses, their ratio is fixed by the sensor layout and does not depend on the strength of the maneuver. A small differential signal therefore increases the variance of $\hat{\kappa }$ but not the error of the reconstructed field. When no maneuver is in progress, ψ and the eddy-current field vanish together and $\hat{\kappa }$ is undefined; $\hat{\kappa }$ is therefore held at one while $∥\psi (t)∥$ remains below a threshold, and the output reduces to the feed-forward value.

The eddy-current field at the primary point is given by the feed-forward reconstruction corrected by the amplitude factor:

(13) $$\hat{b}\left(r_{0},t\right)=\hat{\kappa }\left(t\right)\;{\hat{b}}_{f}\left(r_{0},t\right)$$

Any position within the candidate region is reconstructed using the same expression. The vector-compensated output is $y_{0}-\hat{b}\left(r_{0},t\right)$, and the scalar (total-field) compensation takes its magnitude. Each step consists of the first-order filter recursion of Equation (6), one difference in Equation (8), and one scalar division in Equation (12), so the number of multiply–accumulate operations is on the order of $10^{3}$.

### 3.3. Auxiliary Location and Error Sources

The auxiliary location is chosen as the position at which the eddy-current field differs most from that at the primary point. Because the variance of $\hat{\kappa }$ is inversely proportional to $\psi^{T}R^{-1}\psi$, the feed-forward model is used before installation to compute the RMS value of $∥{\hat{b}}_{f}\left(r_{j},t\right)-{\hat{b}}_{f}\left(r_{0},t\right)∥$ over the maneuver at every feasible mounting position within the candidate region, and the position with the maximum value is selected. This criterion is the single-sensor case of sensor selection on a low-dimensional basis [30].

Apart from measurement noise, four classes of magnetometer error enter the measurement through the following mechanisms. When the axes of the two magnetometers are misaligned by δθ, or when their scale factors differ by δs, the uniform external field leaves a residual on the order of $\delta \theta \;B_{0}\left(t\right)$ or $\delta s\;B_{0}\left(t\right)$ in the difference. The constant part of this residual in the pre-maneuver attitude is removed together with the offset, and the remaining leakage is on the order of $\delta \theta ∥B_{0}\left(t\right)-B_{0}\left(0\right)∥$ or $\delta s∥B_{0}\left(t\right)-B_{0}\left(0\right)∥$, that is, proportional to the change in the geomagnetic vector during the maneuver rather than to its magnitude. A sensor position error δr enters ψ through the local gradient of the modes, with a relative perturbation of approximately $\frac{∥\nabla g∥∥\delta r∥}{∥g∥}$. The dominant effect of an inter-sensor time offset is that the background field no longer cancels in the difference; the remaining term is on the order of $∥{\dot{B}}_{0}∥\left|\delta t_{j}-\delta t_{0}\right|$.

All of these errors enter the result only through the amplitude factor. The four classes of error affect only the estimate $\hat{\kappa }$ in Equation (12). Misalignment and scale-factor errors introduce only a relative error of the same order into the excitation of Equation (8), which likewise enters $\hat{\kappa }$. Because only one unknown is estimated at each sample, adding auxiliary magnetometers does not change the path through which the errors enter the result.

Two further error sources remain. On the measurement side, an external field whose value differs between the primary point and the auxiliary location, such as the field of a ferromagnetic object on the seabed, adds to the difference. Its pre-maneuver value is removed together with the offset. The term $\varepsilon_{a}\left(t\right)$ that remains in the difference is the change in the anomaly difference during the maneuver, produced by the rotation of the body frame and by the translation of the platform through the gradient of the anomaly. It enters $\hat{\kappa }$ as $\frac{\psi^{T}R^{-1}\varepsilon_{a}}{\psi^{T}R^{-1}\psi }$, in the same way as the leakage of a misalignment of $\frac{∥\varepsilon_{a}∥}{∥B_{0}∥}$ rad. On the model side, the errors of the offline model fall into two classes. The amplitude scaling error $\epsilon_{s}$ is absorbed directly by $\hat{\kappa }$. Errors that alter the spatial distribution or the time constants of the modes cannot be absorbed by a single amplitude factor; these are hereafter referred to as shape errors, denoted by $\epsilon_{m}$.

## 4. Simulation Verification

### 4.1. Simulation Design and the Low-Dimensional Mode Library

The verification is based on a simplified solid model of the aluminum-alloy UUV. The model is approximately 7.0 m long, with an outer diameter of approximately 0.53 m and a wall thickness of 8 mm. It retains the conductive load-bearing structures, namely the hull, the stiffening rings, the compartment frames, and the sealed compartments; small local features are simplified, and onboard equipment is not included. The material is 6063-T83 aluminum alloy with an electrical conductivity of $3.03\times 10^{7}$ S/m. The front half of the payload section (approximately 2.0 m) serves as the sensor candidate region. The estimate in Section 2.1 gives $\tau_{\mathrm{est}}\approx 40$ ms, so that Ωτ is approximately 0.014 at π/9 rad/s, or 0.02–0.05 with the effective decay time constants of Table 1. All simulations are three-dimensional transient FEM computations. The seawater domain is treated as air [12], and the problem in the body frame is equivalent to a fixed geometry excited by a time-varying uniform field [10]. The reconstruction error is defined as the ratio of the norm of the error field to that of the true field over all observation points, all three components, and all time samples. The RMS residual at the primary point is the RMS value of the three-component error over all time samples; that is, the eddy-current interference that remains at the primary point after vector compensation.

The FEM setup is as follows. The Magnetic Fields interface of COMSOL Multiphysics 6.1 solves for the magnetic vector potential in the reduced-field formulation. In this formulation, the background flux density $B_{0}\left(t\right)$ is prescribed in the whole domain, and its tangential magnetic field is imposed on the outer boundary of a cubic air domain of 20 m side length. The model contains 34 aluminum domains (hull, stiffening rings, compartment frames, and sealed compartments) and two air domains; the air is given a conductivity of 1 S/m for numerical regularization. The free tetrahedral mesh has 2.91 million second-order vector elements and 18.4 million degrees of freedom. The element size is 1–4.5 cm in the conductors, 3–7 cm in the air near the sensor positions, and up to 1.1 m in the far field. In the conductors, the 8 mm wall is resolved by one layer of second-order elements; the skin depth in the alloy is 29 mm at 10 Hz, so the current density is uniform across the wall. The near-field size settings are a maximum element size of 0.7 m, a minimum size of 0.03 m, a growth rate of 1.35, a curvature factor of 0.3, and a narrow-region resolution of 0.85. Time integration uses the backward differentiation formula of variable order 1–5 with a relative tolerance of 0.01, an initial step of 1 ms, a maximum step of 0.1 s, and steps that end exactly at the output times. The linear systems are solved by a fully coupled biconjugate gradient stabilized (BiCGStab) iteration with a geometric multigrid preconditioner. On a 20-core workstation with 32 GB of memory, one 1 s maneuver solution takes 4.7–7.3 h (30–32 time steps) and one 2 s unit-excitation solution takes 2.6–2.8 days.

The simulation data consist of a unit-excitation set and a maneuver set. In the unit-excitation set, a ramp-and-hold background field is applied along each body axis, rising linearly from 0 to 50 μT during 0–1 s and remaining constant during 1–2 s; this set is used for mode extraction and step-response fitting. The maneuver set consists of the transient solutions of a yaw, a pitch, a roll, and a combined maneuver with a background-field magnitude of 50 μT, and it is used neither for mode extraction nor for step-response fitting. In the yaw, pitch, and roll maneuvers, the angular rate rises linearly from zero to π/9 rad/s within the first 0.1 s and is then held constant, so that the platform rotates through 19° within 1 s. For these maneuvers, 20 time samples are stored at intervals of 0.05 s. In the combined maneuver, the platform rotates about the fixed body-frame axis (0.40, 0.80, 0.45), and the background field is directed along (−0.81, −0.30, 0.51). Its angular rate rises linearly to π/9 rad/s within 0.1 s, is held until 0.7 s, reverses linearly to −π/9 rad/s between 0.7 and 0.9 s, and is held until 1.5 s, so that the rotation angle reaches 14° and returns to 1°. For this maneuver, 30 time samples are stored at intervals of 0.05 s. All three excitation channels of this maneuver are non-zero from the start, with RMS values of 9.9, 8.9, and 7.1 μT/s, and change sign during the reversal, which no single-axis maneuver provides. Additional yaw, pitch, and roll solutions at π/3 rad/s are also available; the yaw and pitch solutions are used for the angular-rate comparison, and all solutions other than the test maneuver are used for the calibration of the T-L model. The data are exported along the longitudinal axis, along an offset line (shifted by 0.1 m in the *y* direction), and on a volume grid covering the candidate region. The RMS value of the eddy-current field at the primary point is 192 nT for yaw, 228 nT for pitch, 195 nT for roll, and 189 nT for the combined maneuver.

An independent model provides a ground truth that differs from the model of the mode library in three respects: the hull conductivity is reduced by 10% to $2.73\times 10^{7}$ S/m, a solid aluminum cylinder is added inside the candidate region, and the geometry is remeshed. The cylinder, of radius 0.12 m and length 0.30 m, lies on the axis at *x* from −1.575 to −1.275 m and acts as an eddy-current source that the mode library does not contain. The remeshed model has 2.92 million elements and 18.5 million degrees of freedom. The yaw maneuver at π/9 rad/s is solved on this model, whereas the mode library, the step responses, and the mode values at the sensor positions remain those of the nominal model. The 126 grid points inside the cylinder are excluded from the region metrics of this test, which use the remaining 2845 air points for both models.

**Figure 1:**
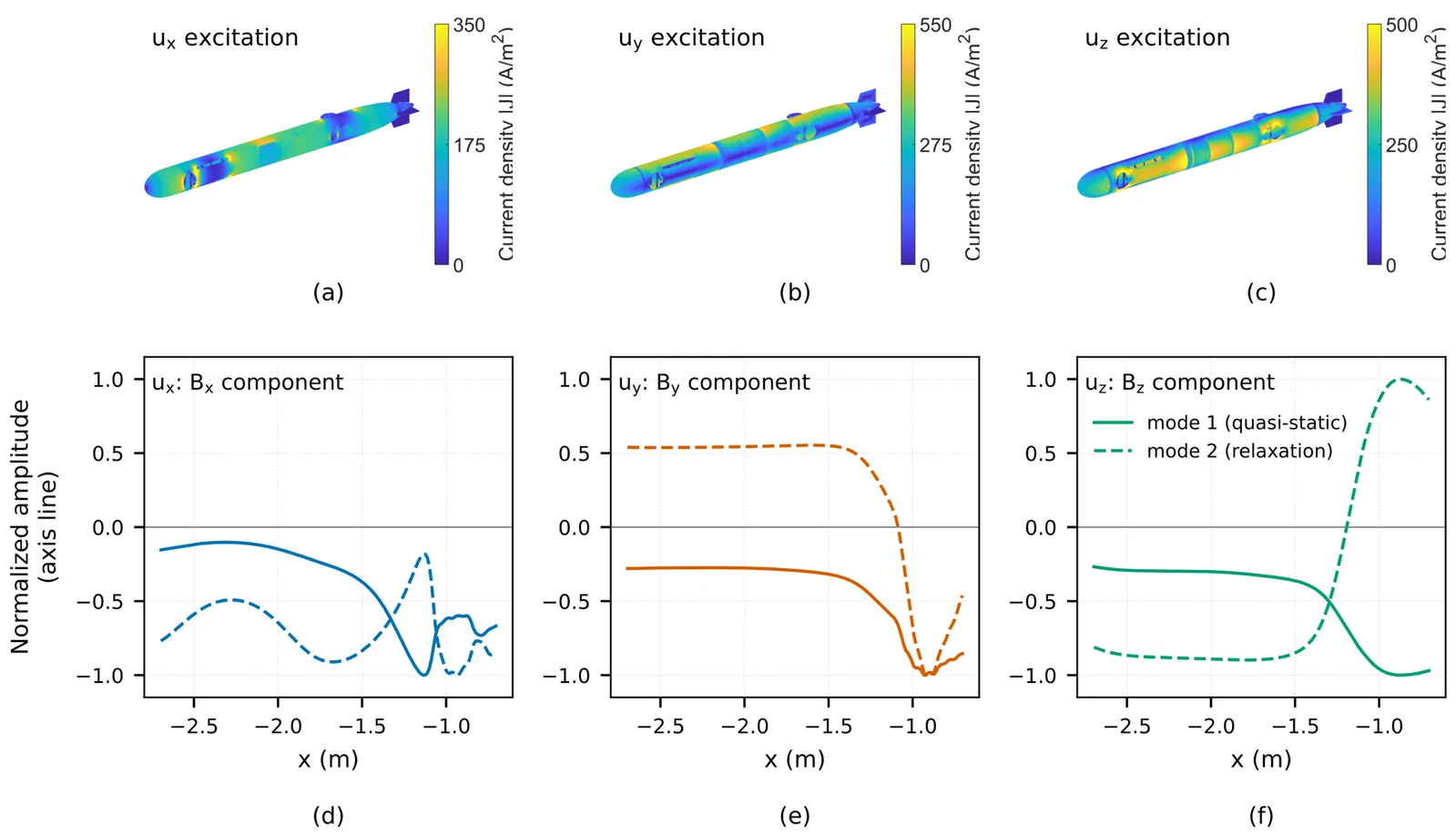
Current density distributions and retained modes under unit excitations along the three body axes. (**a**–**c**) Current density distributions in the conductive structure under the $u_{x}$, $u_{y}$, and $u_{z}$ excitations at *t* = 0.9 s; (**d**–**f**) dominant component of the two retained modes of each excitation channel along the axis line (solid lines: mode 1, quasi-static; dashed lines: mode 2, relaxation).

**Figure 2:**
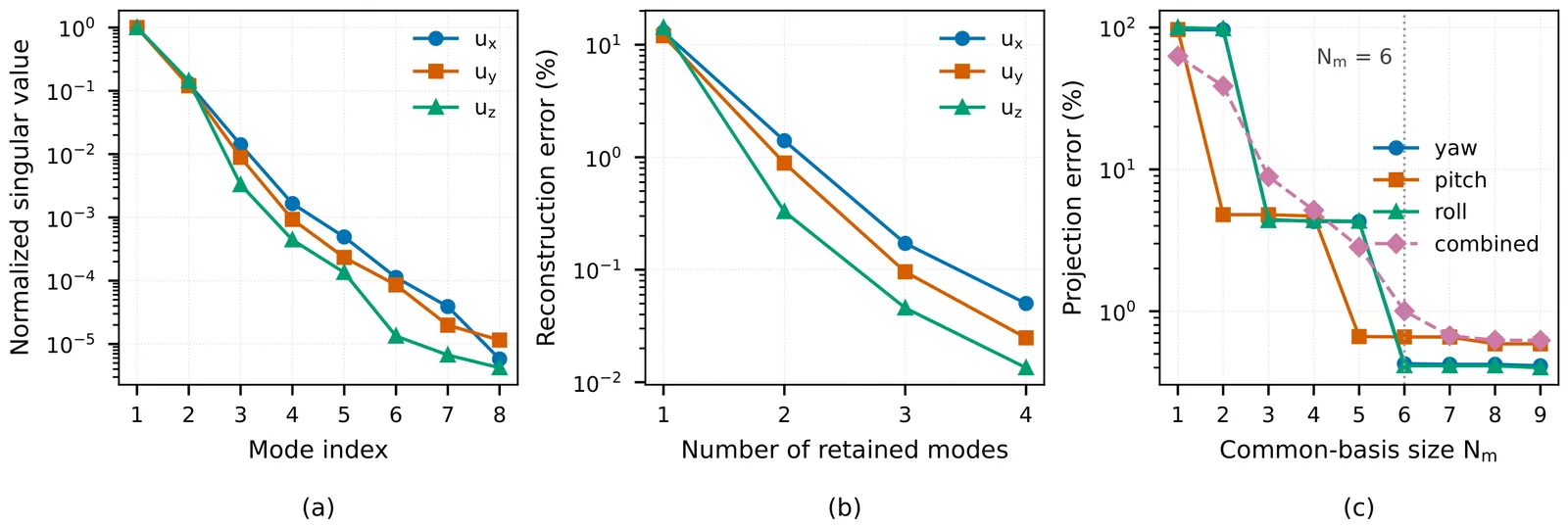
Verification of the mode library: (**a**) normalized singular value spectra of the snapshot matrices for each excitation direction; (**b**) single-direction reconstruction error versus the number of retained modes; (**c**) projection error of the yaw, pitch, and roll maneuvers (π/9 rad/s) and of the combined reversing maneuver onto the common basis of the three directions versus the number of common modes (the vertical dotted line marks $N_{m}=6$).

The unit-excitation solutions determine the number of modes for each excitation direction. The current density distributions and the retained modes under the three unit excitations are shown in Figure 1, and the singular value spectra and reconstruction errors are shown in Figure 2a,b and listed in Table 1. Two modes reduce the single-direction reconstruction error to within 1.4%, and three modes reduce it to within 0.2%. The principal angles between the subspaces of the three directions range from 82.6° to 90.0°, indicating that the modes of different directions are mutually independent.

The mode library passes three independent checks. First, the modes extracted from the ramp stage reconstruct the decay stage, which was not used in the extraction, with an error of no more than 0.8%. Second, the field on the offset line is predicted from the axis-line data alone with an error of 0.08–0.49%. Third, the common basis is projected onto the four maneuver data sets that were not used in the extraction (Figure 2c). With six modes, the reconstruction error on the volume grid of the candidate region is 0.60% for yaw, 0.78% for pitch, and 0.61% for roll, and increasing the number of modes to nine reduces it only to 0.57–0.64%. For the roll solution at π/3 rad/s, the six-mode error is 0.59%. For the combined reversing maneuver, the six-mode error is 1.24% on the volume grid and 1.00% on the two lines. During the constant-rate segments, it is 0.7–0.8%, the same as for the single-axis maneuvers. During the 0.1 s ramp and the reversal, it rises to 3–7%, because the excitation changes rapidly and the single relaxation mode of each channel represents the higher-order relaxation terms less well. Nine modes reduce the overall error to 0.70%. Accordingly, Equation (5) holds with $N_{m}=6$ over the entire candidate region, independently of the angular rate of the maneuver and of the number of channels that are excited simultaneously.

The step responses are taken from the unit-excitation solutions and fitted with multi-exponential functions. The ramp stage provides the step response for 0–1 s, and the decay stage provides a second set of samples through $a\left(t\right)=r\left[S(t)-S(t-1)\right]$. A seven-term exponential fit with time constants of 0.01–0.64 s yields a relative RMS error of 0.3–2.7% for the quasi-static modes and 1.4–7.1% for the relaxation modes. When the exact excitation is substituted into Equation (6) to reconstruct the maneuvers that were not used in the fit, the residual at the primary point is 4.7 nT for yaw and 5.2 nT for pitch, and it is 8–16 nT at the rear of the candidate region, where the eddy-current field is strongest (true values of 400–900 nT). The first-order truncation of Equation (4) is comparable at the primary point but rises to 23–99 nT at the rear of the candidate region.

### 4.2. Sensor Configuration, Error Injection, and Reference Methods

**Figure 3:**
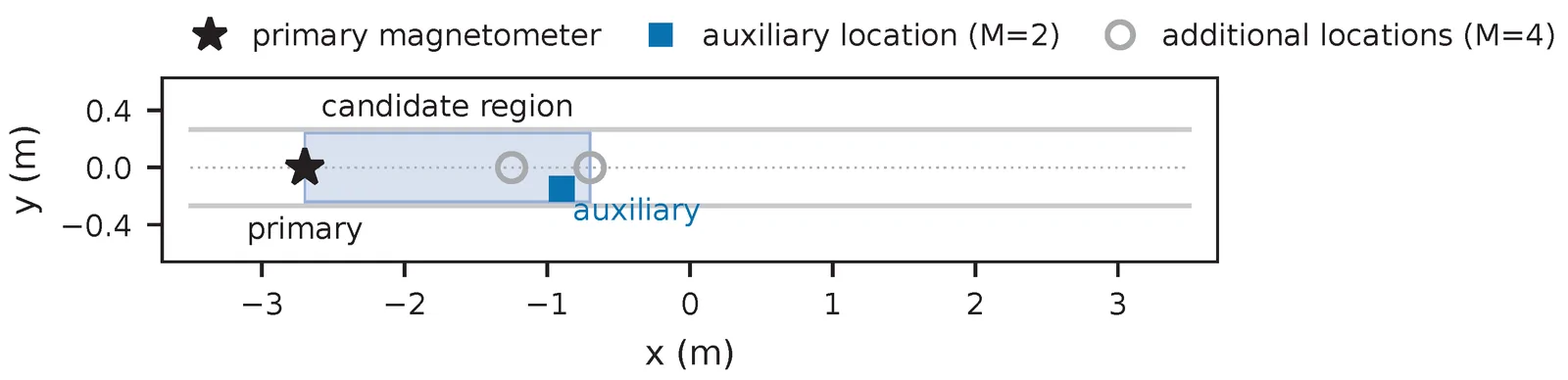
Sensor layout: hull outline, sensor candidate region, primary magnetometer, auxiliary location of the M=2 configuration, and additional locations of the M=4 configuration.

The primary magnetometer and the auxiliary location are configured as follows. The primary magnetometer is placed at the axis-line point (−2.70, 0, 0) m at the front end of the candidate region. Following the criterion of Section 3.3, the RMS difference between the eddy-current field at a grid point and that at the primary point is evaluated over the yaw and pitch maneuvers. Among the 2244 grid points within the candidate region at which a sensor can be mounted, its maximum, 650 nT, occurs at the radial point (−0.90, −0.15, 0) m near the compartment-interface frame at the rear of the candidate region. For comparison, it is 500 nT at the rear end of the axis line and 212 nT at its middle. The configuration of the proposed method is therefore M=2 (Figure 3). Two additional axis-line locations, (−1.25, 0, 0) and (−0.70, 0, 0) m, form the M=4 configuration used for comparison. The roll maneuver is not involved in the location selection and serves as an independent test maneuver; this setting is unfavorable to the proposed method.

The online reconstruction simulations inject errors according to the parameters listed in Table 2. The amplitude scaling error of the model ranges up to ±20% on the basis of the conductivity spread, the temperature effect, and the weld effect. The shape error is generated by applying random perturbations to the unit-excitation snapshots and re-extracting the modes and step responses. Errors are injected on the measurement-generation side, whereas the estimation side uses the nominal model.

We consider four reference methods, all of which share the measurements and the error injection with the proposed method. (i) The T-L model at the primary point [2,18] is used in its complete vector form, $B_{a}=a+A\left[y_{0}\left(t\right)-y_{0}\left(0\right)\right]+C{\dot{y}}_{0}\left(t\right)$, with a permanent term a, a symmetric induced term A, and an eddy-current term C, 18 coefficients in total; the primary magnetometer output serves as the regressor. The induced term absorbs every distortion proportional to the field, including the leakage of the magnetometer scale factor and misalignment after offset removal, and the eddy-current term describes the eddy-current field at the primary point. The coefficients are identified by least squares on the six available maneuvers other than the test maneuver (yaw, pitch, and roll at π/9 and π/3 rad/s and the combined reversing maneuver), with the regressor columns scaled to a unit RMS value. The condition number of the scaled regressor matrix is 6, and it is 13 without the combined maneuver. With only two constant-rate maneuvers, the induced and eddy-current terms are not separable, because ${\dot{y}}_{0}=-\omega \times y_{0}$ within each maneuver, and the condition number exceeds 50; the sensitivity of the eddy-current-only form of the model to the errors of the vector magnetometer is a known issue [38]. Calibration and testing use the same draw of installation errors, which is a setting that favors the T-L model. Because this model does not use the offline model, the two classes of model error do not apply to it; its residual is that of its eddy-current term, and it describes only the calibration point and cannot reconstruct the region. (ii) Open-loop feed-forward is the reconstruction of Equation (6) without the amplitude-factor correction and serves as the ablation reference in which the amplitude factor is removed. (iii) Full-coefficient estimation serves as the ablation reference in which the feed-forward structure is removed, and it is at the same time an online adaptive estimator without a prior on the amplitude. Without using the excitation, it solves for all six coefficients at each sample by weighted least squares from the differential measurement d(t)=Φα(t)+ε(t) of the M=4 configuration, for which the identifiability condition is $3\left(M-1\right)\geq N_{m}$. The condition number of the whitened matrix Φ is 46.5, and its least-squares noise gain at the primary point is 3.26 nT per nT of sensor noise, compared with 0.24–0.27 for the proposed method after the start-up phase. (iv) Adaptive interference cancelation with a pair of magnetometers [32] uses neither an offline model nor attitude information. For each axis, the ratio of the interference at the two sensors is estimated from the correlation of the difference with the two readings, and the interference at the primary point is the difference divided by the ratio minus one. It is applied as published, with the pre-maneuver offset removed and with the whole maneuver as the estimation window.

The methods are compared under three main comparison cases (last row of Table 2). The ideal case contains only 1 nT noise and an exact offline model. The favorable and typical installation cases inject the five classes of magnetometer error and the two classes of model error simultaneously. The magnetometer errors take the magnitudes of a calibrated device and of the datasheet limits, respectively: the favorable installation represents the residual errors after vector calibration, and the typical installation represents an uncalibrated sensor pair or a calibration that has lost validity; both levels are injected into the proposed method and into the reference methods alike. A fourth case, the typical installation with calibrated scale factor and misalignment (0.1% and 0.01°, with the noise, time offset, position error, and model errors of the typical installation), separates the effect of the two calibratable errors from that of the remaining errors. In the favorable and typical installations, $\epsilon_{s}=10\%$ corresponds to the combined magnitude of the temper spread and the temperature effect on the conductivity, and $\epsilon_{m}$ is set to 2% and 5%, respectively. Each case is evaluated with 200 Monte Carlo runs, and the shape error is sampled from 50 perturbed mode libraries with 4 runs each. The evaluation metrics are the region reconstruction error on the volume grid of the candidate region, which is the primary metric, and the RMS residual at the primary point. The yaw, pitch, and roll maneuvers and the combined reversing maneuver serve as test maneuvers with the same sensor configuration.

### 4.3. Online Reconstruction Results

The results for the three main comparison cases, together with the typical installation with calibrated scale factor and misalignment, are listed in Table 3. In the ideal case, the residual of open-loop feed-forward is slightly lower than that of the proposed method, because open-loop feed-forward uses the exact offline model, whereas the proposed method multiplies it by $\hat{\kappa }$, which has not yet converged during the start-up phase. For the complete T-L model, the residual is 8.1–9.5 nT, because its induced and eddy-current terms remain partly collinear (Section 4.2). Once errors are injected, the ranking of the methods changes. Under the favorable installation, the proposed method’s region reconstruction error for yaw, pitch, and roll is 2.7%/3.2%/2.9%, compared with 10.5%/10.7%/9.7% for open-loop feed-forward and 12.0%/8.4%/12.1% for full-coefficient estimation. At the primary point, the residual is 8.0/9.3/8.4 nT for the proposed method and 10.3/9.3/10.4 nT for the T-L model. Under the typical installation, the proposed method yields 6.1%/6.3%/7.3% and 13.6/15.0/15.4 nT, open-loop feed-forward 10.6%/10.9%/9.9%, full-coefficient estimation 45.9%/28.1%/46.3%, and the T-L model 13.1/13.7/13.1 nT. When the scale factor and the misalignment of the typical installation are reduced to their calibrated values, the proposed method yields 3.8%/4.1%/4.1% and 11.1/12.1/11.7 nT, and the T-L model yields 12.6/12.1/ 12.7 nT. The residual of the complete T-L model thus changes little between the typical installation and the calibrated one, because its induced term absorbs the scale-factor and misalignment leakage. When $\epsilon_{s}$ is changed to −10%, the favorable-installation results are comparable, with 2.6%/2.8% and 7.5/7.8 nT for the proposed method and 10.2%/10.1% for open-loop feed-forward (yaw/pitch).

For the combined reversing maneuver (the combined rows of Table 3), the proposed method yields 4.5% and 10.4 nT in the ideal case and 5.0% and 11.6 nT under the favorable installation. Under the typical installation, it yields 5.8% and 14.1 nT, and 5.3% and 13.1 nT with a calibrated scale factor and misalignment. Once model errors are injected, open-loop feed-forward yields 10.3–10.6% and 19.5–21.3 nT, full-coefficient estimation 9.8–26.8% and 32–77 nT, and the T-L model 10.0, 15.0, and 12.5 nT. In the ideal case, the residual of the combined maneuver, 10.4 nT, exceeds that of the single-axis maneuvers because this maneuver contains two transients, as discussed below.

Adaptive cancelation yields residuals of about 5 μT for the single-axis maneuvers and 1.2 μT for the combined maneuver in every case. This is because the ratio of the interference at the two sensors is estimated from readings that are dominated by the change in the geomagnetic vector during the maneuver, which violates the assumption of the method that the interference is uncorrelated with the ambient field. Even if the change in the geomagnetic vector were removed exactly, which would require attitude information, the residual would be 18–24 nT in the ideal case and 30–33 nT under the typical installation with a five-sample window. The reason is that the ratio of the eddy-current field at the two positions varies with time and differs between the axes.

The values in Table 3 are the means of 200 Monte Carlo runs. The run-to-run standard deviation of the residual of the proposed method is 0.1, 1.3–1.8, and 4.8–5.9 nT in the ideal, favorable, and typical cases, respectively, and that of full-coefficient estimation is 0.4–0.6, 8–20, and 40–91 nT; the standard error of the mean is at most 1/14 of the run-to-run standard deviation. No conclusion is drawn from differences smaller than this standard error.

The reference methods are governed by different error sources, as the growth of the residuals across the cases of Table 3 shows. From the ideal case to the typical installation, the residual at the primary point grows by a factor of 2.0–2.2 for the proposed method, 4.8–12 for full-coefficient estimation, and 1.4–1.7 for the complete T-L model. By contrast, the residual of open-loop feed-forward rises by a factor of 3.4–4.4 as soon as the model errors are injected and then changes by less than 6% between the favorable and the typical installation. The error of open-loop feed-forward is set by the amplitude scaling error of the model: $\epsilon_{s}=10\%$ translates directly into a region error of approximately 10%, independently of the installation errors. The error of full-coefficient estimation arises from the representation error of the mode basis at the auxiliary locations and from the sensor errors, both of which are amplified through the inversion, and it is largest under the typical installation. The complete T-L model is insensitive to the scale-factor and misalignment errors, which its induced term absorbs. Its residual of 8–15 nT across the cases is set by the residual collinearity of the induced and eddy-current terms. Adding the combined reversing maneuver to the calibration set lowers the condition number from 13 to 6 and the residual by 0.1–2.0 nT relative to the five constant-rate maneuvers alone, because only a reversal of the angular rate separates the two terms. When the induced term is omitted, as in the eddy-current-only form of the model, the leakage proportional to $B_{0}\left(t\right)$ differs in form from the regressor ${\dot{B}}_{0}$ and cannot be absorbed by the eddy-current coefficients. The residual under the typical installation then rises to 22.1/19.1/19.2 nT with the same six calibration maneuvers and to 35.4/31.8/16.4 nT with two. The errors of the proposed method enter only through the amplitude factor, and the interference is still suppressed by 92–93% under the typical installation. Its margin over open-loop feed-forward in the region error narrows accordingly, from a factor of 3.3–3.9 under the favorable installation to 1.4–1.7 under the typical installation and 2.4–2.8 with calibrated scale factor and misalignment. Relative to the two ablation references, the amplitude factor removes the 10% amplitude error of the model (region error 9.7–10.9% for open-loop feed-forward against 2.7–7.3% once errors are injected). Similarly, the feed-forward structure removes the amplification through the inversion (8.4–46% for full-coefficient estimation against 2.7–7.3%). With the M=4 configuration, the residuals of the proposed method at the primary point in the three cases are 6.6/8.4/7.2, 7.9/9.7/8.5, and 13.2/14.4/14.8 nT. These values differ from those of the M=2 configuration by 1.0 nT or less, in either direction; hence, adding auxiliary locations brings no systematic gain in accuracy.

**Figure 4:**
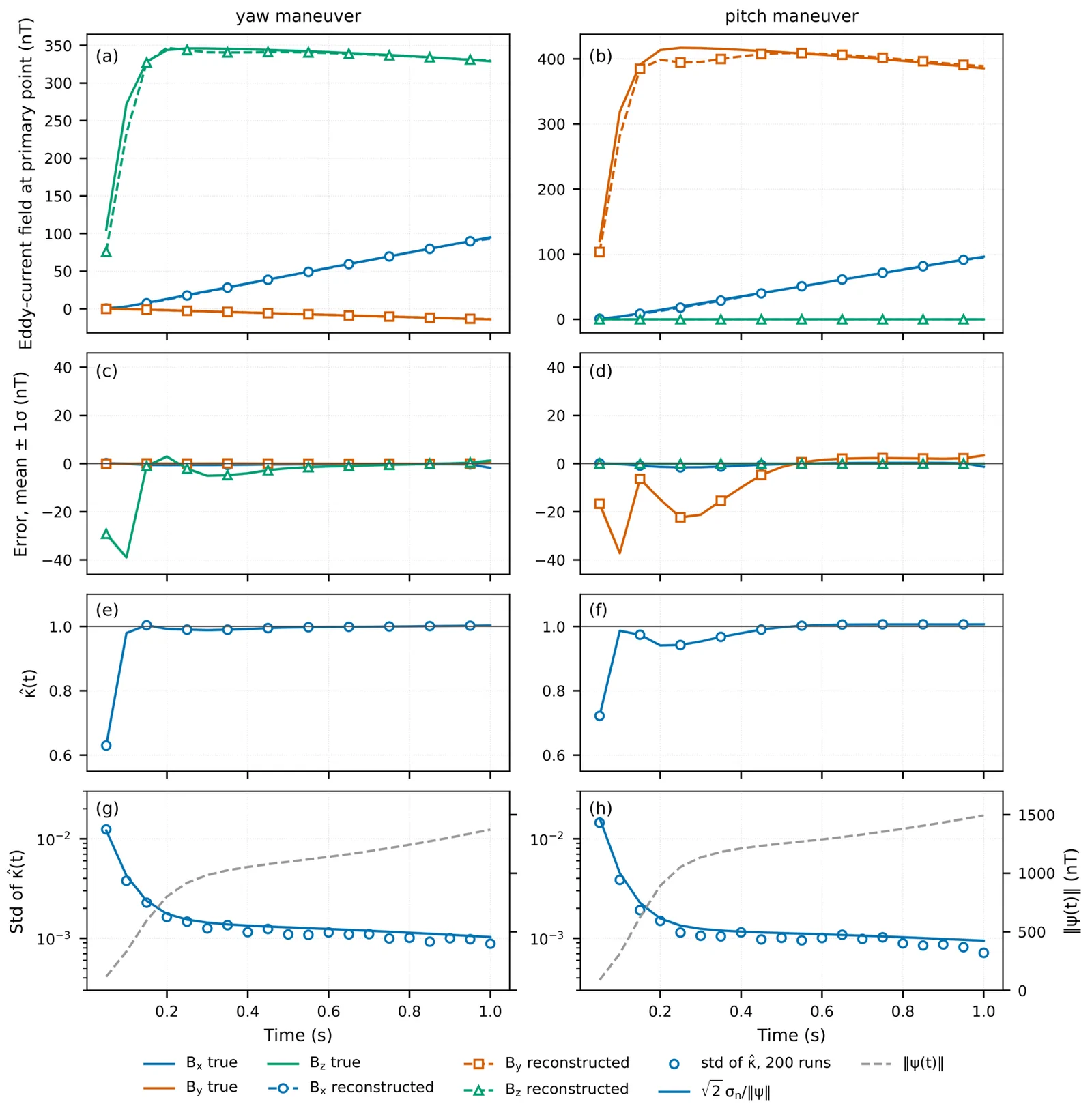
Reconstruction of the eddy-current field at the primary point with the M=2 configuration: (**a**,**b**) time series (solid lines: true values; dashed lines with markers: reconstruction); (**c**,**d**) component-wise errors (lines with markers: mean; shading: ±1 standard deviation); (**e**,**f**) amplitude factor $\hat{\kappa }\left(t\right)$ (line with markers: mean; shading: ±1 standard deviation); (**g**,**h**) standard deviation of $\hat{\kappa }\left(t\right)$ over the runs (circles) and its theoretical value $\frac{\sqrt{2}\;\sigma_{n}}{∥\psi (t)∥}$ (solid line), with the predicted difference $∥\psi (t)∥$ (gray dashed line) on the right axis. The legend entries of the true and reconstructed components refer to (**a**,**b**), and the remaining three entries refer to (**g**,**h**). **Left** column: yaw; **right** column: pitch; ideal case; 200 runs.

**Figure 5:**
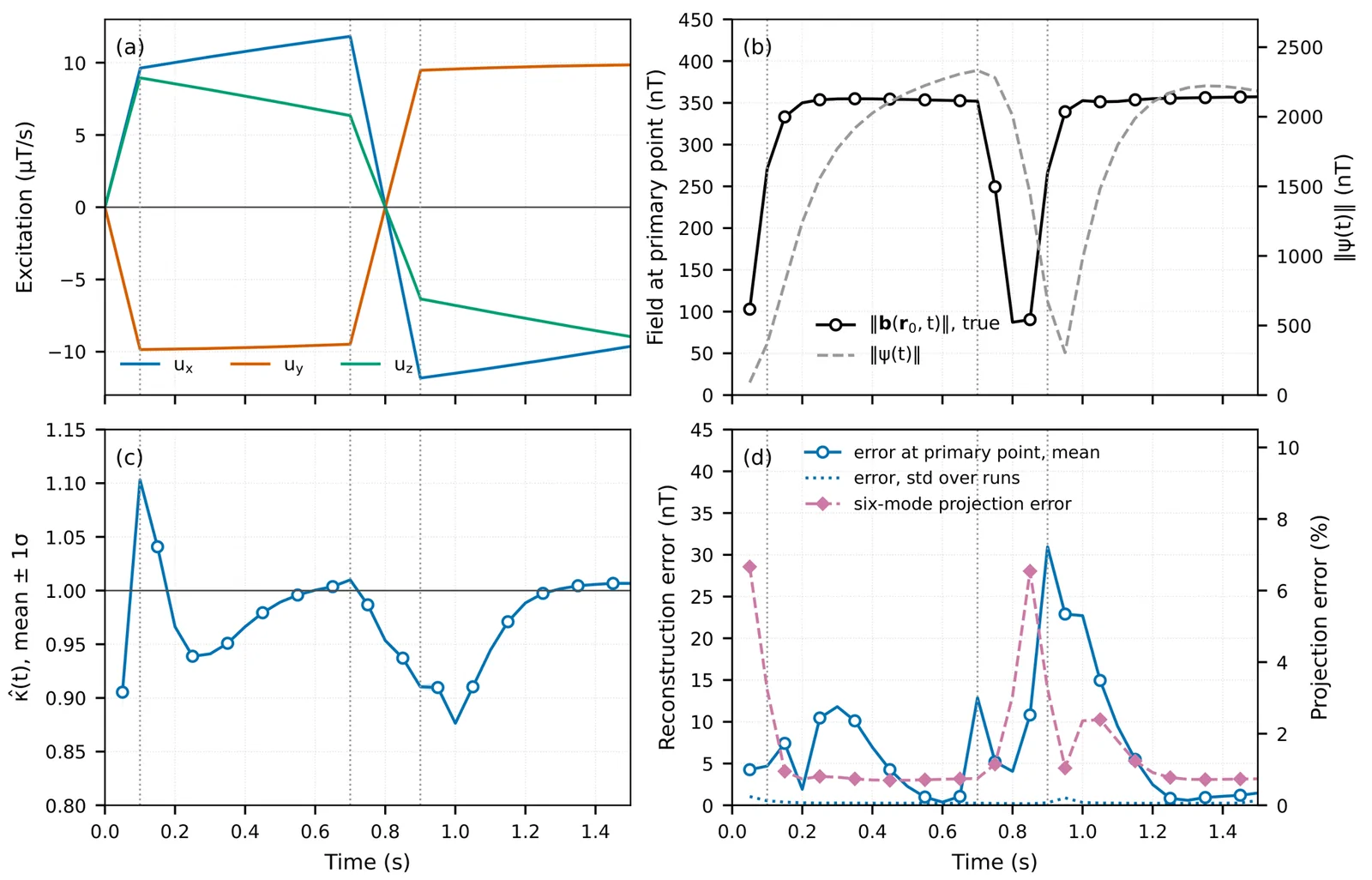
Combined reversing maneuver, ideal case, 200 runs: (**a**) the three excitation channels $u_{x}$, $u_{y}$, and $u_{z}$ obtained from the prescribed background field; (**b**) the magnitude of the true eddy-current field at the primary point and of the predicted difference $∥\psi (t)∥$; (**c**) the amplitude factor $\hat{\kappa }\left(t\right)$, mean ± 1 standard deviation; (**d**) the mean and standard deviation of the reconstruction error at the primary point, together with the projection error of the maneuver data onto the six-mode basis at each time sample. The vertical dotted lines mark the end of the ramp (0.1 s) and the start and end of the reversal (0.7 and 0.9 s).

The convergence of the amplitude factor is shown in Figure 4. In the ideal case, the residual of the proposed method is concentrated within the first 0.3 s after the start of the maneuver, when the eddy-current field has not yet built up, and the amplitude factor is estimated from a small signal. The mean of $\hat{\kappa }$ over the 200 runs stays within 1 ± 0.01 after 0.40 s for yaw and after 0.45 s for pitch and roll. For the combined reversing maneuver (Figure 5), the mean of $\hat{\kappa }$ deviates from unity twice, after the start and after the reversal of the angular rate, and returns to within 1 ± 0.01 in each case 0.55 s after the transient begins. Its standard deviation follows the expression of Section 3.2 through the reversal, where $∥\psi ∥$ passes through a minimum of 300 nT. The standard deviation of $\hat{\kappa }$ over the runs agrees with the value $\frac{\sqrt{2}\;\sigma_{n}}{∥\psi ∥}$ of Section 3.2 at every sample (Figure 4g,h), from 0.012 at 0.05 s, when $∥\psi ∥$ is 90–120 nT, to 0.001 at 1.0 s. The standard deviation of the reconstruction error at the primary point remains below 1.3 nT throughout, whereas the mean error within the first 0.10 s is 17–23 nT. The start-up error is therefore a systematic effect of the mode mismatch rather than a consequence of ill-conditioning. The amplitude factor is estimated sample by sample with Equation (12). In the cumulative form, the start-up samples, for which the model fit is poorest, also enter the estimate, and the residual in the ideal case is higher by 1.1–1.4 nT. Raising the noise from 1 to 10 nT multiplies the standard deviation of $\hat{\kappa }$ by ten but increases the residual by only 1.1 nT (Table 4) and leaves the settling time unchanged. The reason is that Equation (12) solves for a single unknown from three equations at each sample. The start-up phase is the reason why the proposed method trails open-loop feed-forward by 1.5–1.9 nT in the ideal case: 97%, 87%, and 97% of the residual energy of the yaw, pitch, and roll maneuvers falls within the first 0.3 s, and after 0.3 s the residual is 1.3, 3.2, and 1.5 nT, at the level of open-loop feed-forward. For the combined maneuver, 95% of the residual energy falls within the two 0.5 s intervals that follow the start and the reversal; the residual outside these intervals is 4.4 nT, and it is 1.2 nT in the final constant-rate segment. The mode projection error of the maneuver data rises to 3–7% during the same intervals (Section 4.1), so the deviation of the amplitude factor follows the mode mismatch rather than the noise. For maneuvers longer than those simulated here, the share of the transients in the total residual decreases accordingly. We tested three treatments of the start-up phase. The first regularizes Equation (12) with the prior $\kappa ∼N(1,0.05^{2})$ derived from the error budget. The second sets $\hat{\kappa }=1$ while the predicted difference $∥\psi (t)∥$ remains below 200 nT; this is the threshold rule of Section 3.2, which is not applied in Table 3. The third uses the cumulative form while $∥\psi (t)∥$ remains below 100–300 nT and then switches to the sample-by-sample form. In the ideal case, the first two treatments change the residual by −0.8 to +0.3 nT, and under the favorable installation, the second one raises the pitch residual by 1.4 nT; the third treatment leaves the residual unchanged, and none of them closes the gap. The gap is caused by the mode mismatch at the auxiliary location during the build-up of the eddy current rather than by noise.

**Figure 6:**
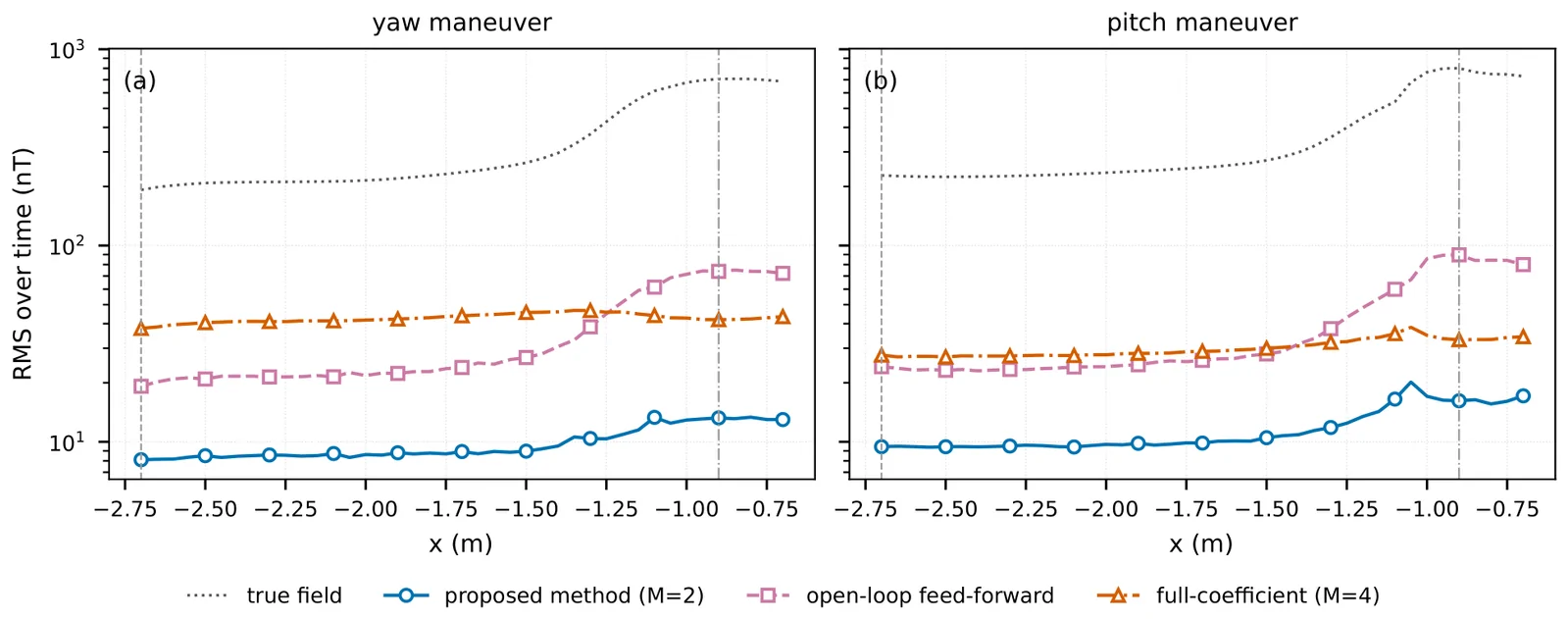
Point-wise RMS reconstruction residual along the longitudinal axis (y=z=0) under the favorable installation for the proposed method, open-loop feed-forward, and full-coefficient estimation; the dotted line denotes the RMS value of the true field. (**a**) Yaw; (**b**) pitch. The vertical dashed line marks the primary point, and the dash-dotted line marks the longitudinal position of the auxiliary location; 200 runs.

The error distribution within the region is shown in Figure 6 for yaw and pitch. Under the favorable installation, the point-wise RMS residual of the proposed method along the longitudinal axis is 8.1–13.4 nT for yaw and 9.4–20.2 nT for pitch. It does not grow with the true field, which rises to 400–900 nT at the rear of the candidate region. The median of the point residuals on the volume grid is 8.8/9.9/9.3 nT, and the maximum is 40/38/37 nT, both of the same order as the residual at the primary point; the region error is lower than the relative residual at the primary point because this metric is dominated by the high-field part of the region. Along the longitudinal axis, the residual of open-loop feed-forward is 19–90 nT and that of full-coefficient estimation is 27–47 nT; under the typical installation, the median of full-coefficient estimation rises to 164/94/165 nT, which indicates that the error amplified through the inversion spreads over the entire region.

The proposed method requires no recalibration when the primary magnetometer is relocated. When the primary magnetometer is moved to the axis-line point (−1.70, 0, 0) m in the middle of the candidate region, where the eddy-current interference is 237/250 nT, with the auxiliary location and the offline model left unchanged, the residual under the favorable installation is 9.6/10.0 nT. The T-L model calibrated at the original primary point and transferred directly to the new position yields 39.6/24.7 nT, and 13.5/11.4 nT after recalibration at the new position. The T-L model requires calibration maneuvers covering all three axes at every installation position and cannot be extrapolated to other positions. In the ideal case, the scalar compensation residual at the primary point is 0.6 nT, compared with an RMS total-field eddy-current interference of 10.1/20.3 nT before compensation.

### 4.4. Error Budget and Robustness

**Figure 7:**
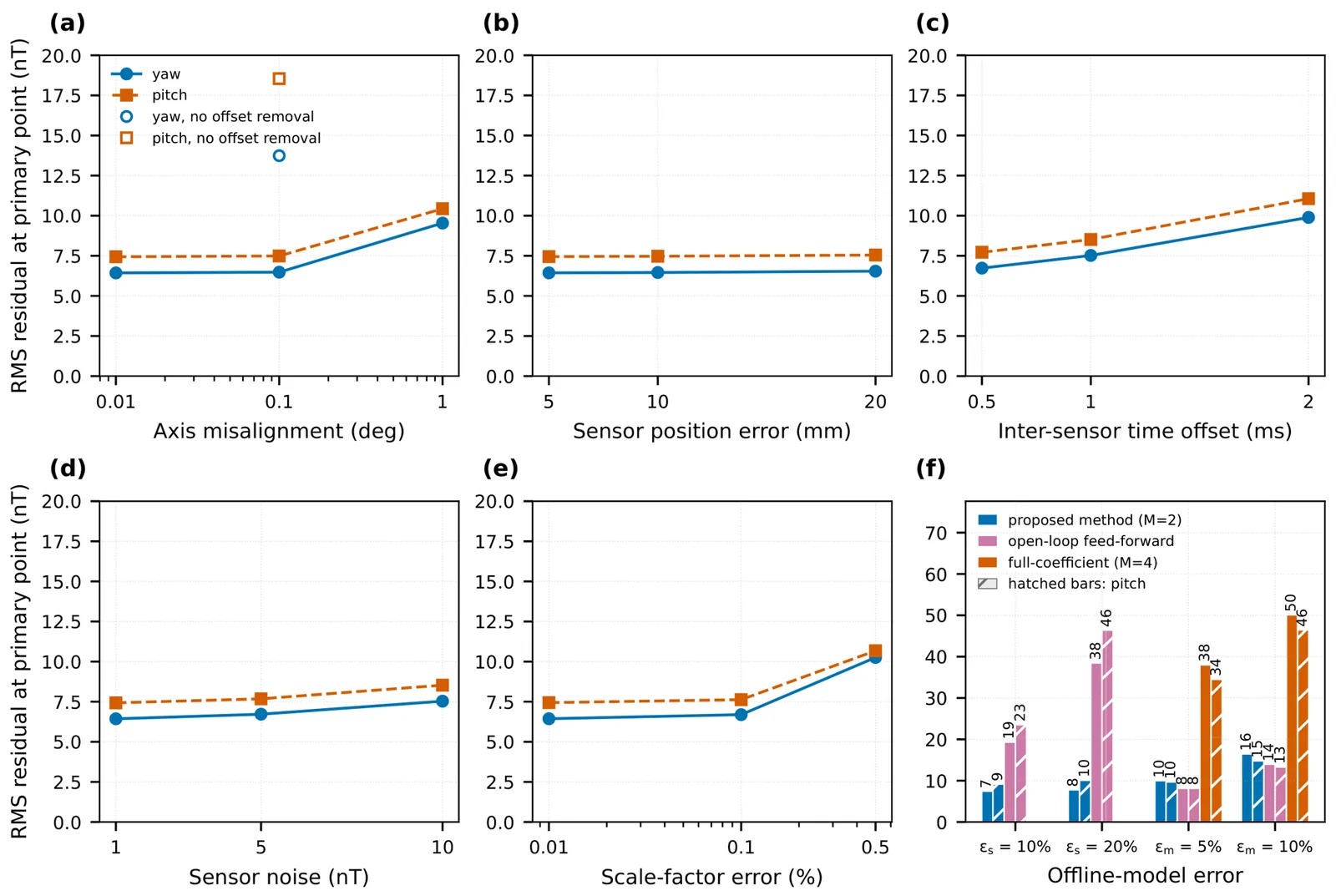
Robustness tests (M=2, RMS residual at the primary point): (**a**) axis misalignment (open markers: without offset removal); (**b**) position error; (**c**) inter-sensor time offset; (**d**) noise; (**e**) scale factor (in (**a**–**e**), solid lines with circles: yaw; dashed lines with squares: pitch); (**f**) residuals of the proposed method, open-loop feed-forward, and full-coefficient estimation under the model errors $\epsilon_{s}$ and $\epsilon_{m}$ (hatched bars: pitch; full-coefficient estimation is unaffected by $\epsilon_{s}$ and is therefore not plotted).

The individual effects of the five classes of magnetometer error are listed in Table 4 and shown in Figure 7a–e, with 200 runs per level, the other errors set to zero, and a noise level of 1 nT. A misalignment of 1° and a scale-factor error of 0.5% each increase the residual by approximately 3 nT, a position error of 20 mm changes it by less than 0.1 nT, and an inter-sensor time offset of 2 ms increases it by approximately 4 nT. For a rotation angle of 19° and a single-factor residual limit of 10 nT, the corresponding tolerances are approximately 1° of axis misalignment, 0.5% of scale factor, and 2 ms of inter-sensor time offset. Because the leakage of misalignment and scale-factor errors is proportional to the rotation angle, these tolerances tighten as the rotation angle increases.

Offset removal accounts for the wide tolerances of misalignment and scale-factor errors. After offset removal, these two errors leak only through the change in the geomagnetic vector. For a rotation of 19°, the RMS value of $∥B_{0}\left(t\right)-B_{0}\left(0\right)∥$ is approximately 10 μT, so that 0.1° of misalignment or 0.1% of scale-factor mismatch corresponds to a differential leakage of approximately 17 nT or 10 nT, that is, 1.5–2.6% of the 650 nT differential signal at the auxiliary location. Without offset removal, the residual for 0.1° of misalignment is 13.7/18.5 nT.

The separate effects of the two classes of model error are shown in Figure 7f, with the other errors set to the favorable-installation values. For amplitude scaling errors of $\epsilon_{s}=\pm 10\%$ and ±20%, the proposed method yields 6.9–7.4/7.4–9.1 nT and 6.7–7.7/6.7–10.0 nT, open-loop feed-forward yields 19.3–20.7/23.5–24.0 nT and 38.4–39.8/46.4–46.7 nT, and full-coefficient estimation is unaffected. The shape error cannot be absorbed by a single amplitude factor: for $\epsilon_{m}=5\%$ and 10%, the proposed method yields 9.9/9.6 and 16.4/14.7 nT, open-loop feed-forward 8.1/8.1 and 13.9/13.2 nT, and full-coefficient estimation 38.0/34.5 and 50.1/46.4 nT. When the shape error acts alone, the residual of open-loop feed-forward is slightly lower than that of the proposed method, because the amplitude factor is fitted at the auxiliary location, and the shape error there is carried to the primary point through $\hat{\kappa }$. When amplitude and shape errors coexist, the residual of the proposed method remains below that of open-loop feed-forward (Table 3). The condition under which the proposed method outperforms open-loop feed-forward is that the amplitude scaling error exceeds the shape error: at $\epsilon_{s}=5\%$, open-loop feed-forward already rises to 10.2/12.5 nT, whereas at $\epsilon_{m}=5\%$ the proposed method yields 9.9/9.6 nT, so the crossover point lies at approximately 5% for both errors. According to the budget in Table 2, the combined magnitude of $\epsilon_{s}$ exceeds 10%, so this condition is satisfied; if calibration on a real platform has reduced $\epsilon_{s}$ to below 5%, the two methods yield comparable residuals.

The independent model of Section 4.1 provides a ground truth whose deviation from the offline model is not prescribed. Its eddy-current field is 0.90 times the nominal one at both magnetometer positions, and the best scalar fit of the nominal field leaves a shape residual of 1.2% at the primary point and 2.0% in the difference at the auxiliary location. Over the volume grid, the projection error of the true field onto the nominal six-mode basis is 15.7%. Almost all of it comes from the grid points within 0.125 m of the added cylinder (28.6% there against 1.9% elsewhere), because the near field of the cylinder is not contained in the basis. With the nominal model on the estimation side, the amplitude factor converges to 0.899. The residual at the primary point is 4.2 nT with 1 nT noise and 5.1/9.8/6.6 nT with the magnetometer errors of the favorable, typical, and calibrated typical installations and no injected model error. On the nominal truth, the corresponding values are 6.4/7.0/10.9/8.1 nT. Open-loop feed-forward yields 18.9 nT, because the 10% amplitude deviation remains uncorrected. The residual is lower on the perturbed truth than on the nominal one because the eddy-current field of the perturbed model is 10% weaker and relaxes faster. Its ratio to the nominal field at the primary point is 0.96 at 0.05 s and 0.90 from 0.3 s onward. The start-up error is therefore smaller (12 nT against 17–23 nT within the first 0.10 s). The region error is 15.8–16.8% over all 2845 air points but 2.5/3.0/6.3% (ideal, favorable, typical) away from the cylinder, close to the 2.0/2.5/5.6% obtained on the nominal truth. Full-coefficient estimation, however, fails on this model (residual above 330 nT), because its auxiliary location at (−1.25, 0, 0) m lies 2.5 cm from the end of the cylinder and its reading is dominated by a field outside the basis. A localized source therefore affects the reconstruction only in its own neighborhood, and the shape error it produces at the magnetometer positions is of the order of the 2% perturbation level of Table 2. A shape error spread over the whole structure, which the random perturbations of the mode library represent, is a separate case.

The residual increase under the typical installation can be decomposed by error source. Relative to the ideal case, the residual increase for yaw and pitch is 7.2/7.6 nT. The single-factor increase is 3.9/3.3 nT for a 0.5% scale-factor error, 2.8/1.4 nT for a 5% shape error, 1.0–1.7 nT each for a 1 ms time offset and a 10% amplitude error, and less than 0.5 nT in total for noise, misalignment, and position error. The sum of the single-factor increases, 9.3/8.0 nT, is slightly larger than the combined increase, which indicates that the error sources are partly independent. Reducing the scale-factor error from 0.5% to 0.1% by calibration is the single measure that yields the largest gain.

We quantify the effect of an external anomaly by superposition. A static magnetic dipole fixed in the world frame is placed below the middle of the candidate region, and the platform travels along its longitudinal axis at 2 m/s while performing the maneuver. The field of the dipole is added to the readings of both magnetometers. This superposition is valid because the aluminum hull has a relative permeability of one and does not alter a static external field. This was verified with a stationary FEM solution of the hull model in which a uniformly magnetized sphere (dipole moment 167 A·m^2^) is placed 3 m below the hull axis. After the image effect of the outer boundary of the air domain (2% at 10 m) is accounted for, the computed field agrees with the analytical dipole field to 0.4% at both magnetometer positions and over the candidate region. The difference between the two positions also agrees to 0.4%. In addition, the eddy-current response to the rate of change of the anomaly at the primary point is added to the truth with the nominal model. After offset removal, the term $\varepsilon_{a}$ of Section 3.3 is the change in the anomaly difference between the two positions during the maneuver. Table 5 lists the results in the ideal case for dipoles whose field at the primary point is 100–5000 nT at depths of 5–20 m below the hull axis. The increase in the residual is at most 0.16 times the RMS value of $\varepsilon_{a}$. Pitch is the most sensitive maneuver, because the rotation about the *z* axis turns the vertical anomaly field into the *x* component, which is the mechanism of a misalignment leak. For yaw and roll, the change is produced mainly by the translation through the anomaly gradient. The speed dependence confirms this attribution for the 1000 nT anomaly at 5 m. From 0 to 4 m/s, the yaw residual grows from 6.8 to 46.1 nT and the roll residual from 8.6 to 13.5 nT. The pitch residual, however, is already 54.9 nT at rest, and its two contributions partly cancel at 1 m/s (14.9 nT) before adding up at 2 and 4 m/s (59.6 and 74.8 nT). A horizontal dipole at 2 m/s yields 26–28 nT for all three maneuvers. Overall, the residual increases by less than 3 nT (5 nT for pitch) as long as the RMS value of $\varepsilon_{a}$ does not exceed 50 nT. This condition corresponds to an anomaly of 1000 nT at a distance of at least 20 m, of 100 nT at 5 m, or of 5000 nT at 50 m. Geological anomalies with gradients below 10 nT/m satisfy this condition, whereas a compact ferromagnetic object within 10 m does not. Such cases are detected from the part of the differential measurement that Equation (10) does not explain. Its RMS value is 1–4 nT without an anomaly and exceeds 20 nT in every case of Table 5 in which the residual increases by more than 3 nT. When an anomaly is expected, κ can be estimated jointly with a linear drift of the difference over the samples after 0.5 s. This removes most of the translation-induced bias (for the 1000 nT anomaly at 5 m, the yaw residual decreases from 28 to 9 nT) at the cost of 0.3–1.3 nT without an anomaly. This joint estimation is not adopted as the default.

The conclusions hold when the angular rate is increased to π/3 rad/s. The eddy-current interference then rises to 578/638 nT. The region reconstruction error of the proposed method in the ideal, favorable, and typical cases is 1.6%/2.2%, 2.0%/2.6%, and 3.7%/4.0%, the residual at the primary point is 11.7/19.2, 14.6/23.7, and 27.8/34.7 nT, and the suppression ratio is 95–98%. Open-loop feed-forward yields 9.7%/10.7% under the favorable installation, and full-coefficient estimation yields 40.7%/27.3% under the typical installation.

## 5. Conclusions

We have proposed a real-time reconstruction method for the eddy-current magnetic field. A low-dimensional spatial basis and its step responses are extracted offline; online, the output of the primary magnetometer drives a feed-forward reconstruction, and a single auxiliary magnetometer corrects its amplitude. The method has been verified with transient FEM simulations, and the main conclusions are as follows.

(1) For low-frequency maneuvers and linear materials, the eddy-current field of the platform is spanned by six maneuver-independent modes, namely a quasi-static mode and a relaxation mode for each body-axis excitation channel. These modes represent the yaw, pitch, and roll maneuvers, which were not used in the mode extraction, over the entire sensor candidate region with a reconstruction error below 0.8%. They also represent a combined reversing maneuver in which the three channels act simultaneously with an error of 1.2% (0.7–0.8% during the constant-rate segments and 3–7% during the two transients). The time response of each mode coefficient is represented by its step response in multi-exponential form; with the exact excitation, the feed-forward reconstruction error is approximately 2%, and the first-order truncation holds only where the relaxation component is weak.
(2) The rate of change of the primary magnetometer output can serve as the excitation, and the amplitude deviation of the offline model is corrected sample by sample with one amplitude factor obtained from the differential measurement of a single auxiliary magnetometer. For maneuvers at π/9 rad/s with the five classes of magnetometer error and the two classes of model error injected simultaneously, the region reconstruction error within the candidate region is 2.7–3.2% and the residual at the primary point is 8–9 nT under a favorable installation, and the corresponding values are 6.1–7.3% and 14–15 nT under a typical installation. For the combined reversing maneuver, they are 5.0% and 12 nT and 5.8% and 14 nT. Under the same conditions, the region error of open-loop feed-forward is 9.7–10.9%, and that of full-coefficient estimation is 8.4–46%. When the primary magnetometer is moved to another position without recalibration, the residual is 10 nT. An independent FEM model of modified conductivity, an added conductor, and a new mesh is also used as the ground truth, while the method keeps the original offline model. In this test, the amplitude factor converges to 0.90, the true ratio between the fields of the two models, and the residual at the primary point remains at 4–10 nT. The region error rises only within 0.125 m of the added conductor, whose near field is not contained in the mode basis.
(3) Magnetometer errors enter the result only through the amplitude factor. For a single-factor residual of no more than 10 nT, the tolerances are approximately 1° of axis misalignment, 0.5% of scale factor, and 2 ms of inter-sensor time offset. A ±20% amplitude error of the offline model changes the residual by less than 3 nT, and a 10% shape error raises it to 15–16 nT. At its calibration point, the complete T-L model identified from the six other maneuvers is comparable to the proposed method, with 8–10 nT in the ideal case and 13–15 nT under a typical installation over the four test maneuvers. Its induced term absorbs the scale-factor and misalignment leakage. Its residual rises to 25–40 nT when the model calibrated at the original position is used at a new position. Moreover, the separation of its induced and eddy-current terms requires calibration maneuvers about all three axes, at more than one angular rate and with a reversal of the angular rate, at every installation position. The advantage of the proposed method therefore lies in the region reconstruction, the relocation without recalibration, and the absence of calibration maneuvers rather than in the accuracy at the calibration point.

These conclusions were obtained for linear materials, low-frequency maneuvers, and FEM models of a single platform. Six aspects remain to be addressed. First, shape-type model errors were examined with randomly perturbed mode libraries and with one independent FEM model. The near field of a conductor that the mode library does not contain degrades the reconstruction in its own neighborhood, so the non-uniform conductivity of a real platform and the eddy-current sources of onboard equipment require a correction of the mode basis from measured data. On a real platform, the excitation in Equation (8) is also affected by the induced field, which varies with attitude and must be handled within the calibration procedure. Second, the combined maneuver verified here rotates about one fixed oblique axis for 1.5 s; maneuvers about time-varying axes and longer maneuver sequences remain to be verified. Third, the mode mismatch at the auxiliary location during the build-up of the eddy current causes the amplitude factor to deviate within the first 0.3 s of a maneuver and within 0.55 s after each reversal of the angular rate. Neither prior regularization nor threshold switching removes this deviation. The planned remedy is to extend the grid of time constants of the multi-exponential fit of the step responses of the relaxation modes below 0.01 s and to verify whether the feed-forward error at the auxiliary location during the build-up decreases. Fourth, the treatment of misalignment drift between the two offset removals and experiments on a real platform remain to be carried out. Fifth, an external field that differs between the two magnetometer positions, such as that of a ferromagnetic object within 10 m of the hull, biases the amplitude factor when its change during the maneuver exceeds about 50 nT. Such cases are detected from the unexplained part of the differential measurement, and the reconstruction then falls back to the feed-forward value. Sixth, the online algorithm was run at the 20 Hz storage rate of the FEM solutions; at the higher sampling rates of real magnetometers, the differencing of Equation (8) and the start-up behavior remain to be verified.

## Figures and Tables

**Table 1:** Number of modes and reconstruction error for each excitation direction (axis line and offset line, payload section).

Excitation Direction	1 Mode	2 Modes	3 Modes	Effective Decay Time Constant (s)
*x*	13.0%	1.40%	0.17%	0.09–0.14
*y*	12.0%	0.89%	0.10%	0.065–0.074
*z*	14.2%	0.33%	0.046%	0.069–0.086

**Table 2:** Simulation parameters, error budget, and sources.

Item	Value	Source
Hull conductivity	6063-T83, $3.03\times 10^{7}$ S/m, or 52% of the International Annealed Copper Standard (IACS); 50%–58% IACS across tempers (±8%); temperature coefficient 0.39%/°C, ±10 °C corresponds to ±4%	[36]
Weld seams, contact resistance, geometric simplification	The simplified model overestimates the measured field; analytical and finite element method (FEM) results differ by less than 20%	[12]
Model amplitude scaling error $\epsilon_{s}$/shape error $\epsilon_{m}$	0, ±5%, ±10%, ±20%/random snapshot perturbation of 2%, 5%, 10%; independent FEM model (conductivity × 0.9, added conductor, new mesh)	Combination of the two rows above/this work
Magnetometer scale factor	0.01%, 0.1%, 0.5% (datasheet <±0.5%; 0.1% after calibration)	[37]
Magnetometer axis misalignment	0.01°, 0.1°, 1° (datasheet orthogonality <0.5°, calibrated version <0.1°)	[37]
Noise, position error, inter-sensor time offset	1/5/10 nT, 5/10/20 mm, 0.5/1/2 ms	This work
Main comparison cases	Ideal: 1 nT noise, exact model; favorable installation: 0.01°, 5 mm, 1 nT, 0.5 ms, 0.1%, $\epsilon_{s}=10\%$, $\epsilon_{m}=2\%$; typical installation: 0.1°, 10 mm, 5 nT, 1 ms, 0.5%, $\epsilon_{s}=10\%$, $\epsilon_{m}=5\%$	The items above

**Table 3:** Region reconstruction error (volume grid of the candidate region, %) and root-mean-square (RMS) residual at the primary point (nT) under the three main comparison cases and the typical installation with calibrated scale factor and misalignment. Each cell lists yaw/pitch/roll; the combined rows list the region error/residual of the combined reversing maneuver. The region error is an energy-weighted metric dominated by the high-field part of the region. The RMS eddy-current interference at the primary point is 192/228/195 nT, and 189 nT for the combined maneuver. The Tolles–Lawson (T-L) model (complete form, calibrated on the six other maneuvers) and adaptive cancelation provide no region reconstruction.

Case	Metric	Proposed Method (M=2)	Open-Loop Feed-Forward	Full-Coefficient (M=4)	T-L Model	Adaptive Cancelation
Ideal	Region (%)	2.0/2.5/2.2	1.9/2.1/2.3	3.0/4.9/3.2	—	—
Ideal	Primary point (nT)	6.4/7.4/7.1	4.9/5.5/5.6	9.6/16.6/10.2	9.3/8.1/9.5	5153/5198/5119
Ideal, combined	Region (%)/point (nT)	4.5/10.4	2.5/6.6	6.1/22.1	—/8.9	—/1175
Favorable installation	Region (%)	2.7/3.2/2.9	10.5/10.7/9.7	12.0/8.4/12.1	—	—
Favorable installation	Primary point (nT)	8.0/9.3/8.4	19.1/24.1/18.9	32.2/26.4/32.5	10.3/9.3/10.4	5151/5197/5117
Favorable, combined	Region (%)/point (nT)	5.0/11.6	10.3/19.5	9.8/31.9	—/10.0	—/1173
Typical installation	Region (%)	6.1/6.3/7.3	10.6/10.9/9.9	45.9/28.1/46.3	—	—
Typical installation	Primary point (nT)	13.6/15.0/15.4	19.7/25.5/19.5	115.1/80.4/115.6	13.1/13.7/13.1	5142/5191/5107
Typical, combined	Region (%)/point (nT)	5.8/14.1	10.6/21.3	26.8/76.7	—/15.0	—/1175
Typical, calibrated	Region (%)	3.8/4.1/4.1	10.6/10.9/9.9	17.0/14.0/16.9	—	—
Typical, calibrated	Primary point (nT)	11.1/12.1/11.7	19.7/25.6/19.5	54.2/45.4/53.8	12.6/12.1/12.7	5151/5197/5117
Typical, calibrated, combined	Region (%)/point (nT)	5.3/13.1	10.6/21.3	16.0/51.9	—/12.5	—/1173

**Table 4:** Magnetometer error sweeps (RMS residual at the primary point, nT, yaw/pitch; each cell gives “error level: residual”; the other errors are zero, and the sensor noise is 1 nT).

Error	Level 1	Level 2	Level 3
Axis misalignment (after offset removal)	0.01°: 6.4/7.4	0.1°: 6.5/7.5	1°: 9.5/10.4
Scale factor (after offset removal)	0.01%: 6.4/7.4	0.1%: 6.7/7.6	0.5%: 10.3/10.7
Position error	5 mm: 6.4/7.5	10 mm: 6.5/7.5	20 mm: 6.5/7.5
Noise	1 nT: 6.4/7.4	5 nT: 6.7/7.7	10 nT: 7.5/8.5
Inter-sensor time offset	0.5 ms: 6.7/7.7	1 ms: 7.5/8.5	2 ms: 9.9/11.1

**Table 5:** Effect of an external anomaly (ideal case, speed 2 m/s, vertical dipole below the middle of the candidate region): RMS change in the anomaly difference during the maneuver, RMS residual at the primary point, and RMS value of the unexplained part of the differential measurement (all in nT); each cell lists yaw/pitch/roll. Without an anomaly, the residual is 6.4/7.4/7.1 nT, and the unexplained part is 4.0/4.1/1.3 nT.

Depth (m)	Anomaly at the Primary Point (nT)	Change in the Anomaly Difference	Residual	Unexplained Part
5	100	45/34/44	6.8/10.4/7.1	25/8/25
5	1000	450/336/445	28.3/59.6/10.3	239/61/255
10	1000	128/57/126	8.0/8.8/7.6	72/32/72
10	5000	642/285/632	29.0/15.3/14.8	358/163/362
20	1000	33/52/33	6.5/9.3/7.2	20/19/18
20	5000	166/261/165	7.5/29.5/9.4	95/107/93

## Data Availability

The exported finite element field data and the processed results supporting the findings of this study are openly available in the Science Data Bank at https://doi.org/10.57760/sciencedb.space.0032o (accessed on 27 September 2026) and in the Supplementary Material. The finite element model files (approximately 116 GB in total) are available from the corresponding author on request because of their size.

## Supplementary Materials

The following supporting information can be downloaded at: https://www.mdpi.com/article/10.3390/s26216945/s1, File S1: UUV_eddy_current_minimal_dataset.zip (exported finite element field data, processed result files, and a README describing every file).
